# Seroprevalence of Crimean-Congo Hemorrhagic Fever Virus, Bulgaria

**DOI:** 10.3201/eid1901.120299

**Published:** 2013-01

**Authors:** Iva Christova, Teodora Gladnishka, Evgenia Taseva, Nikolay Kalvatchev, Katerina Tsergouli, Anna Papa

**Affiliations:** Author affiliations: National Center of Infectious and Parasitic Diseases, Sofia, Bulgaria (I. Christova, T. Gladnishka, E. Tasseva, N. Kalvatchev);; Aristotle University of Thessaloniki, Thessaloniki, Greece (K. Tsergouli, A. Papa)

**Keywords:** Crimean-Congo hemorrhagic fever, CCHF, CCHF virus, seroprevalence, Bulgaria, viruses

**To the Editor:** Crimean-Congo hemorrhagic fever (CCHF) is endemic in southern Russia, southeastern Europe, Africa, the Middle East, and southwestern Asia (*1*). The incidence and spread of the disease have increased in recent years. In Bulgaria, located on the Balkan Peninsula, CCHF is endemic. The disease was first described in the country in 1952 (*2*). Since then, a mandatory reporting system has been introduced. Most of Bulgaria is an ecologically favorable environment for CCHF virus (CCHFV) circulation in nature. In the 1970s, numerous virologic and serologic studies were performed by Vasilenko et al., who showed that the most affected age group was 21–50 years and that most of those with CCHF were male (65%) (cited in [*3*]). A genetic study showed that CCHFV strains in Bulgaria cluster together with strains from other Balkan countries and Russia (*2*). A vaccine consisting of chloroform-inactivated CCHFV was developed in 1974, and the currently used vaccine strain, isolated from a Bulgarian patient, was characterized genetically (*4*).

In the last 10 years, <10 CCHF cases have been registered annually in Bulgaria. Although the number of cases is lower than previously, the disease has spread into new areas (southeast, northeast, south-central provinces). In 2008, a cluster of cases was observed in southwestern Bulgaria (Blagoevgrad district), a low-risk CCHF area (*5*). Since then, a substantial number of cases have been reported in this district. During the past 4 years (2008–2011), 30 CCHF cases have been registered in Bulgaria, 12 from Blagoevgrad district, 8 from Burgas district, 4 each from Haskovo and Sliven districts, and 1 each from Kardjali and Shumen districts.

To estimate the current situation on CCHFV seroprevalence in both disease-endemic and -nonendemic areas in Bulgaria, we tested serum samples for CCHFV IgG antibodies using a commercially available ELISA kit (Vector Best, Novosibirsk, Russia). The serum samples were collected prospectively during 2011 from 1,018 healthy persons (50.2% male) from 13 districts: Sofia (n = 116), Blagoevgrad (n = 100), Pazardjik (n = 52), Stara Zagora (n = 36), Smolyan (n = 46), Yambol (n = 60), Haskovo (n = 108), Kardjali (n = 50), Sliven (n = 50), Burgas (n = 200), Shumen (n = 50), Ruse (n = 100), and Pleven (n = 50); they were then tested for CCHFV IgG antibodies with a commercially available ELISA kit (Vector Best). The median age of participants was 48 years (range 2–89 years). Persons previously vaccinated against CCHFV were excluded from the study.

Twenty-eight persons (2.8%) had IgG antibodies to CCHFV. The highest seroprevalence was observed in Burgas (7.6%), followed by Kardjali (6%), Pazardjik (5.8%), and Haskovo (4.6%) districts (Figure). Low seroprevalence levels were detected in Sliven (2%), Blagoevgrad (1%), and Ruse (1%) districts. Generally, these results are consistent with the number of reported cases in different districts. Notably, Kardjali and Pazardjik districts showed high CCHFV seroprevalence but single reported cases in the last years. However, these regions were among the main endemic foci in the past. In contrast, the low seroprevalence rate found in district of Blagoevgrad conflicts with the high number of diagnosed CCHF cases, but this district has been at low risk for many years.

**Figure:**
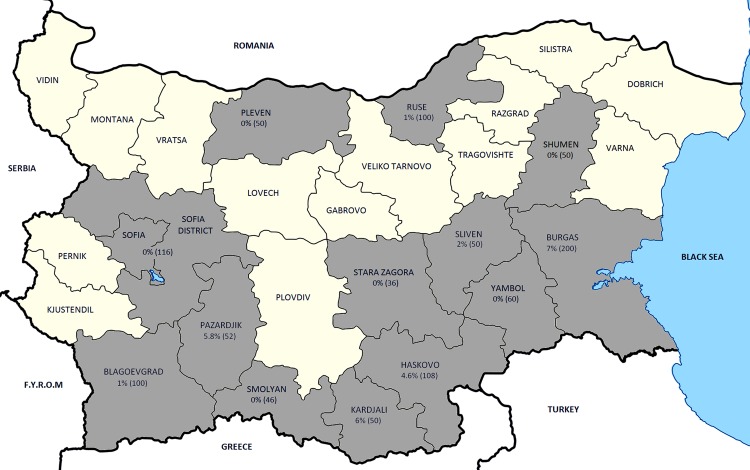
Prevalence rates for Crimean-Congo hemorrhagic fever virus in various districts of Bulgaria. F.Y.R.M., Former Yugoslav Republic of Macedonia.

Multivariate analysis showed that having a former tick bite and farming were significant risk factors, while age and sex were not related to seropositivity (Table). Although no significant difference was seen among age groups, none of the samples from persons 0–19 years of age were seropositive, whereas seroprevalence levels were increasing in those 20–59 years (2.65%) and 60–89 years (3.37%). This increase would be expected because the probability of contacting the virus increases with age. The main risk factor for the 20–29 year age group was the tick bite, and farming and contact with animals were incriminated in the older age groups.

**Table:** Univariate and multivariate regression analysis of CCHFV seropositivity in human population, Bulgaria*

Variable	No. (%) IgG positive, n = 28	No. (%) IgG negative, n = 990	Univariate analysis			Multivariate analysis	
			OR (95% CI)	p value		OR (95% CI)	p value
Age, y			1.00 (0.99–1.03)	0.494			
Median	46	48					
Range	20–83	2–89					
Sex				0.456			
M	16 (3.1)	495 (96.9)	1.33 (0.62–2.85)				
F	12 (2.4)	495 (97.9)	Ref				
Tick bite				<0.001			<0.001
Yes	15 (9.3)	147 (90.7)	6.62 (3.09–14.19)			5.40 (2.47–11.84)	
No	13 (1.5)	843 (98.5)	Ref				
Animal contact				0.253			
Yes	11 (3.7)	290 (96.3)	1.56 (0.72–3.38)				
No	17 (2.4)	700 (97.6)	Ref				
Farming				0.001			0.012
Yes	13 (6.7)	182 (93.3)	3.85 (1.80–8.23)			2.76 (1.25–6.08)	
No	15 (1.8)	808 (98.2)	Ref				

*CCHFV, Congo-Crimean hemorrhagic fever; OR, odds ratio; Ref, reference.

A similar study conducted in Greece, a neighboring country, showed an overall seroprevalence of 4.2%; slaughtering and agricultural activities were significant risk factors for CCHFV seropositivity (*6*). Notably, the seroprevalence levels in the Greek districts Rodopi and Evros (4.95% and 4.49%, respectively) were similar to those in neighboring Bulgarian districts Kardjali and Haskovo (6% and 4.6%, respectively).

We found that the risk for seropositivity was increased 5.4-fold in persons bitten by ticks. Increased tick aggressiveness in years that have favorable climatic conditions results in high rates of attacks on humans and an increased number of tick-borne diseases (*7*). A recent survey for CCHFV in ticks in Haskovo, Kardzhali, and Stara Zagora districts showed that 4.83%, 2.09%, and 1.46%, respectively, were infected by CCHFV, and that the most infected tick was *Hyalomma marginatum* (*8*). These results coincide with those of the current study because Kardzhali and Haskovo were among the districts with the highest seropositivity.

Because of the increasing spread of CCHFV in new foci, public health awareness of this problem is essential. Studies giving information about the spread and ecology of the virus can provide the necessary data for risk assessment analysis and even for prediction of epidemics.
